# Improvements in practising nurses’ knowledge, skills, self-efficacy, confidence, and satisfaction after a simulated clinical experience of caring for a patient undergoing chemotherapy: a quasi-experimental study

**DOI:** 10.1186/s12912-024-01727-0

**Published:** 2024-01-24

**Authors:** Jefferson Garcia Guerrero, Dena Marwan Attallah, Nada Hassan Gomma, Samah Abdulwahed Ali

**Affiliations:** 1 College of Nursing, King Khalid University, Abha Al-Qureiger Campus, 62529 Abha, Saudi Arabia; 2 Nursing Department - Nursing Program, Fakeeh College for Medical Sciences, Abdul Wahab Naib Al Haram, Al-Hamra’a, 23323 Jeddah, Saudi Arabia; 3 Ain Shams University, Faculty of Nursing, Al Mohamady, Al-Waili, Cairo Governorate, Egypt, Egypt

**Keywords:** Simulation modalities, High-fidelity simulation, Virtual simulation, Self-efficacy, Satisfaction, Professional competence, Nurses, Quasi-experimental study

## Abstract

**Background:**

The beneficial effect of simulation experience on nursing students is well established in the literature. However, an accurate simulation modality to help professional nurses enhance their clinical competence and expertise remains unexplored. The current study evaluated and contrasted the impact of two simulation modalities on nurses’ knowledge, abilities, self-efficacy, confidence, and satisfaction following a simulated clinical experience caring for chemotherapy patients.

**Methods:**

A quasi-experimental research design was employed in this study. The participants were divided into group A, comprising nurses exposed to the high-fidelity simulation, and group B, comprising nurses exposed to the virtual simulation.

**Results:**

The study found that nurses exposed to high-fidelity simulation and virtual simulation gained a high standard of knowledge and skills. The nurses’ post-test and post-objective structured clinical examination (OSCE) scores drastically increased after simulation exposure compared to their pre-test and pre-OSCE scores. For the group exposed to high-fidelity simulation, the mean differences were − 19.65 (pre- and post-test) and 23.85 (pre- and post-OSCE), while for the group exposed to virtual simulation, the mean differences were − 22.42 (pre- and post-test) and 20.63 (pre- and post-OSCE). All *p*-values indicated significant differences < 0.001. Moreover, both groups exhibited high self-efficacy, confidence, and satisfaction levels after the simulation experience. The outcomes of both simulation modalities regarding self-efficacy, confidence, and satisfaction levels indicate no significant difference, as supported by *p*-values of > 0.05.

**Conclusion:**

High-fidelity simulation and virtual simulation training effectively and efficiently advance nurses’ professional competence. The nurses exposed to high-fidelity simulation and virtual simulation gained high levels of knowledge and skills. Additionally, it increased their sense of happiness, self-worth, and self-efficacy. The simulation approach will be a potent instrument for improving nurses’ competency and fully developing their sense of expertise. Therefore, developing policies adopting simulation as part of their professional development will ensure patient safety and improve health outcomes.

**Supplementary Information:**

The online version contains supplementary material available at 10.1186/s12912-024-01727-0.

## Background

Chemotherapy is an essential treatment for many types of cancer [1]. Therefore, the expertise of nurses in caring for patients undergoing chemotherapy is vital. Their important responsibilities include gathering the patient’s health history, assessing their physical and psychological status, reviewing treatment plans with the oncologist, ensuring accurate delivery of the chemotherapy, and monitoring the outcomes and possible complications of the treatment [2]. Additionally, providing nursing care to cancer patients also requires sensitivity [3–5], holistic care, and respect for patients’ preferences and needs [3, 6]. To ensure the implementation of quality cancer patient care, a specialized training is necessary for professional nurses to gain specific knowledge and skills including a positive attitude towards collaborative cancer care [7].

However, there is still a shortage of trained oncology nurses [8] and nurses without specialized oncology training face difficulties in providing cancer care due to inadequate education, skills, and competence [9, 10]. Additionally, 16.9% of 402 nurses working in a cancer care facility were exposed to skin and eye cytotoxic drugs [11, 12] and 12% of 2069 nurses experienced cytotoxic spill due to mistaken attachment and detachment of intravenous administration set to the chemotherapy bag during preparation, with 10% of that cytotoxic spill not cleaned up [12, 13]. Nurses also felt incompetent and lacked professional confidence to prepare patients, administer chemotherapy drugs, and monitor patients during chemotherapy [14, 15]. For that reasons, nurses’ education and training in dealing with chemotherapy patients is essential to ensure safe and quality care [12, 16]. Furthermore, simulation-based learning (SBL) is considered a powerful tool to achieve this goal in enriching and boosting nurses’ competency [17]. However, the most effective simulation modality for training professional nurses is still unknown.

Moreover, simulation is widely used among nursing students to enhance their knowledge, competence [18], and skills in clinical settings [19]. During their exposure to the simulation scenario, nursing students can commit every possible medical error in a safe environment without harming patients [20]. Simulation-based learning (SBL) provides the most effective use of cognitive load that improves learning outcomes [21], increases psychomotor skills acquisition and retention [22], triggers comprehension, and decreases unpleasant emotions [23]. Additionally, SBL has been found to improve students’ communication skills [24], problem-solving abilities [25], and teamwork and collaboration [26]. Learners can interact and empathize with simulated patients in a well-developed scenario to improve their positive attitudes [27]. Furthermore, SBL provides supportive feedback and advances learning [28]. Chang et al. [29] stated that simulation bridged the gap between theory and practice.

Nevertheless, according to Hung et al. [30], research conducted to measure varying outcomes of SBL has been limited, and the types of SBL remain undefined. The impact of simulation exposure and the identification of an accurate simulation modality on professional nurses to help them enhance their clinical competence and expertise remains unclear. Furthermore, no evidence exists to confirm whether simulation exposure can enhance their knowledge, skills, self-efficacy, confidence, and satisfaction.

A high-fidelity situation (HFS) is a healthcare education method that utilises sophisticated manikins that simulate human physiology in realistic patient environments [31]. According to the Healthcare Simulation Dictionary, a virtual simulation (VS) recreates a realistic scenario on a computer screen [31]. Guerrero et al. [32] showed that repeated HFS exposure enhances proficiency and competency among nursing interns. Furthermore, exposure to HFS improves knowledge, skills, performance [33], and self-efficacy among nursing students [34]. Additionally, it boosts professional nurses’ self-confidence and provides a high level of satisfaction in learning [17]. VS also positively impacts the learning of nursing students and is effective for refining knowledge and confidence, self-efficacy in communicating with patients, and satisfaction with learning [35–37]. Moreover, self-efficacy is effective in gaining knowledge and enhancing professional skills [38]. Bandura [39] specified that individuals with high levels of self-efficacy display more assertiveness when dealing with problems, and self-efficacy boosts those cognitive resources and drive of an individual that influence their level of control in a particular situation. Furthermore, Guerrero et al. [40, 41] reported that both simulation modalities (HFS and VS) are essential for improving nurses’ professional competence.

HFS provides a safe environment in real-time to determine enacted nursing actions that challenge the participants’ clinical judgment [42]. Furthermore, simulation experience can also improve critical thinking skills [43]; increase the levels of knowledge, satisfaction, and self-confidence [44]; and have positive effects on self-efficacy [45]. However, some disadvantages of HFS include the limited number of practising simulation educators and the time-consuming efforts required to create an authentic clinical scenario and prepare the human-patient simulator and labs [42]. Moreover, VS training improves knowledge, clinical reasoning, self-efficacy, competency, and confidence [46, 47]. However, the video game license that is used for VS can be expensive depending on the simulation type, graphics, and interface [48]. Additionally, user interface issues such as language barriers, software navigation, and software virtual presentation were identified as disadvantages of VS [49]. Thus, the advantages of HFS and VS are limited to nursing students, and the evidence supporting the outcomes for both simulation modalities in professional nurses is scarce.

The four phases of Kolb’s Experiential Learning Theory, such as abstract idea, concrete understanding, active investigation, and reflection, are one of the theories of SBL. To acquire an exclusive experience, participants undergo a simulated case or situation, participate in reflective sessions via debriefing of the case or situation, determine and minimise performance gaps, and conceptualise training. The Experiential Learning Theory is a key strategy to reduce the gap between theory and practice. Thus, integrating simulation into nursing education effectively [50] prevents skill deterioration [51].

Employing evidence-based strategies such as simulation is needed to prepare professional nurses for safe clinical practice [28]. State-of-the-art simulation technology can be utilised to assess clinical skill acquisition, competence, and knowledge development [40, 51]. Simulation also improves self-efficacy [30], confidence, and satisfaction among nursing students [17]. Moran et al. reported the same findings [52], provided that the simulation session was well-planned. Additionally, competency, self-efficacy, and learning satisfaction are some of the major advantages of SBL [53]. However, most prior studies focused on undergraduate nursing students and excluded professional nurses.

## Methods

### Aim of the study

This study aimed to evaluate and compare two simulation modalities—HFS and VS—and their effects on professional nurses’ knowledge, skills, self-efficacy, confidence, and satisfaction through a simulated clinical experience involving caring for patients undergoing chemotherapy.

### Design

This study employed a quasi-experimental experimental design. The knowledge, skills, self-efficacy, confidence, and satisfaction levels of professional nurses were evaluated by dividing them into groups A (exposed to HFS) and B (exposed to VS).

### Settings

The HFS sessions were conducted at the Clinical Skills and Simulation Centre (CSSC) of Fakeeh College for Medical Sciences (FCMS) and Dr. Soliman Fakeeh Hospital (DSFH) using the two high-fidelity simulators belonging to the institutions—CAE Healthcare’s Apollo and iStan with CAE LearningSpace—to document and record the scenarios. The critical care and medical-surgical simulation laboratories were utilised during the training. Each laboratory accommodated 30 participants per day, with five participants in each session. Additionally, in two hospital theatres, VS sessions were conducted utilising the software application Body Interact. Thirty people were allotted to each theatre per day.

### Participants

All participants worked as bedside nurses at the DSFH. Among the 350 nurses employed at the hospital, 256 were qualified to participate in this study according to the criteria set by the researcher. The study participants included Saudi and non-Saudi nationals aged between 27 and 48 years and comprised men (11) and women (109) working in the medical-surgical unit, critical care unit, and outpatient department of DSFH. We intentionally selected nurses from varied hospital settings and considered nurses without experience in the oncology unit to avoid any bias from the prior knowledge and skills (experience) of nurses already working in the Oncology unit. All participants had a two-year diploma in nursing or a bachelor’s or master’s degree in nursing with the same patient-care roles and responsibilities, had a license to practice nursing in Saudi Arabia, and generally had more than one year of experience, thus ensuring their prior knowledge of and experience in patient care. Finally, the participants had no prior experience involving simulation from their undergraduate program to their present status to avoid all biases on their performance and outcomes. Furthermore, nurses who had participated in previous studies comparing the impacts of HFS and VS on nurses’ knowledge, skills, confidence, and satisfaction were excluded from the current study.

In total, 120 nurses were finally selected. Next, 60 participants each were allocated to group A (nurses exposed to HFS) and group B (nurses exposed to VS). The researchers randomly allocated the participants using their employee numbers via a paper lottery system. The recommended sample size was 64 nurses for each group (A and B) based on a G*Power calculation with a 5% margin of error, 95% confidence level, and 80% test power. However, only 120 nurses consented to participate in this study.

### Data collection

The following three similar scenarios and objectives related to caring for a patient undergoing chemotherapy were used for both simulation modalities (HFS and VS): (1) preparation and assessment of patients for chemotherapy, (2) preparation and administration of chemotherapy drugs, and (3) managing patients during and after chemotherapy. The HFS training used a human-patient simulator, while the VS training utilised the BodyInteract app. The study was conducted from August to September 2022 at the Dr. Soliman Fakeeh Hospital and Fakeeh College for Medical Sciences– Clinical Skills and Simulation Center.

Before the study began, participants in Group A initially underwent orientation regarding the planned activities. Pre-briefing, simulation scenarios, and debriefing were observed during the HFS sessions. The success of the simulation and participant learning relies on feedback and reflection during debriefing [41]. Four facilitators were present in each laboratory for each HFS session. Facilitators started the pre-briefing sessions by providing simulation instructions, discussing the scenarios and learning objectives, assigning roles and tasks, and showing the simulation environment, including the equipment needed for the scenario. This pre-briefing lasted 45–50 min and included answering the participants’ questions. The simulation scenario ended after 8–13 min. After the session, the participating nurses were debriefed using the GAS debriefing model for 30–40 min. Each scenario was conducted daily for three days, and both simulation modalities were run simultaneously in separate locations. All simulation facilitators for both modalities also played the role of debriefing facilitators. They were trained to facilitate debriefing using a three-phased debriefing (GAS debriefing model) and multiphase debriefing (Healthcare simulation after action review [AAR] framework) structures since the simulation centre of the institution was established.

The participants in Group B underwent a one-hour technical skills session to learn more about the program’s strengths. Another hour of practice rounds was required after the participants had downloaded the BodyInteract app on their phones. During the VS sessions, three facilitators were present in each theatre to address the participants’ concerns in case of technical difficulties, emergencies, or any other situation that may arise. Facilitators also conducted pre-briefing, VS scenarios, and debriefing sessions. Participants first attended a pre-briefing session about the simulation lasting 20–30 min, where they were informed about the experiment and their learning objectives. The virtual simulation scenario lasted 7–12 min, and there were minimal interactions with the simulation facilitators regarding technical issues. After the simulation, participants were debriefed in person for 30–40 min according to the gather, analyse, and summarise (GAS) debriefing model.

The pre-test was administered to participants before beginning each simulation scenario, and the post-test was administered immediately after the simulation sessions ended. The test questions were prepared and selected by the simulation facilitators prior to the sessions from the Advanced Oncology Nursing Certification Review and Resource Manual 2nd Edition [54]. The questions included 15 multiple-choice questions with five choices and three open-ended essay questions. Prior to the study, 10 nursing faculty members were recruited for the pilot study to ensure that the difficulty index of each examination question was appropriate, and the test did not exceed 30 min. The difficulty index of a question was categorised as very easy, easy, intermediate, difficult, and hard. The pilot study had three easy, eight intermediate, two difficult, and two hard questions. The exam had a reliability score of 0.83 using Cronbach’s alpha, with an alpha value of 0.73–0.95 indicating high reliability [55]. The test was administered using a software called Speedwell, installed on the institution’s iPads.

The simulation facilitators also prepared the objective structured clinical examination (OSCE) scenarios and rubrics according to the references [56, 57] used in the HFS and VS scenarios. The rubric indicators relied on the procedure that was measured by a 4-point Likert-type scale ranging as follows: *done correctly* (3), *done incompletely* (2), *done incorrectly* (1), and *not done* (0). The same test questions and OSCE rubrics were administered during the pre- and post-tests and the pre- and post-OSCE. Additionally, the proctors during the pre- and post-tests and the raters during the pre- and post-OSCEs were blinded to the identities of the nurses under investigation. Another 10 nurses were invited to a mock OSCE using the checklist to ensure that every OSCE station could be performed in under 5 min. The OSCE results had a reliability score of 0.81 using kappa coefficients. A kappa result of 0.81–1.00 indicates almost perfect agreement [58]. The pre-OSCEs were conducted immediately after the pre-tests before the simulation sessions on the same day. All pre-OSCEs were conducted without providing feedback to assess the current skills of nurses regarding the scenarios. However, all post-OSCEs were conducted with feedback.

After the HFS sessions, all participating nurses answered the Student Satisfaction and Self-confidence in Learning (SSS) questionnaire from the National League for Nursing [59]. The tool contains 13 items for assessing nurses’ attitudes regarding their satisfaction with the simulation experience. The five items labelled *satisfaction with current learning* were used to assess the nurses’ satisfaction with the instruction approach, learning equipment diversity, support, reinforcement, and overall manner of the simulation session. Another eight items labelled *self-confidence with learning*, were used to measure participants’ confidence in expertise, the need for the simulation content, improvement of the technique, and understanding of how to identify clinical quandaries in the simulation sessions. The Likert-type choices to answer these items included *strongly disagree* (5), *disagree* (4), *undecided* (3), *agree* (2), and *strongly agree* (1). A reliability of 0.94 on the satisfaction subscale and 0.87 on the self-confidence subscale was recorded using Cronbach’s alpha [60]. Results were calculated through the computation of responses and higher scores that confirmed greater satisfaction and confidence.

The General Self-Efficacy Scale [61] was also used to evaluate the nurses’ positive personal beliefs about managing various complex demands from the simulation experience. This scale contains 10 items on a self-reporting standard of self-efficacy. The Likert-type choices to answer these items included *exactly true* (4), *moderately true* (3), (2) *hardly true* (2), and (1) *not at all true* (1). The total score ranges from 10 to 40, with a higher score signifying high self-efficacy [61]. Cronbach’s alpha was recorded to be between 0.76 and 0.90. The validity of this scale relates to feelings, optimism, and work satisfaction.

### Data analysis

Means and standard deviations (SD) were used to summarise the gathered data. A paired t-test was conducted to compare the nurses’ pre- and post-test and pre- and post-OSCE scores. An independent samples t-test was conducted to compare the effect of the variables on the groups exposed to HFS and the other group exposed to VS. The *p*-values below 0.05 indicated statistically significant differences. Moreover, IBM SPSS Statistics 20 was used to analyse the data.

### Ethical considerations

Approval from the Fakeeh College for Medical Sciences Institutional Review Board (Approval No. 286/IRB/2022) was obtained before the study commenced, and a written informed consent form was given and signed by each participant. The purpose of the study was explained to the participants, including their rights to withdraw. The participants were assured that their privacy was protected, and all information obtained was kept confidential. Furthermore, all methods were performed in accordance with the Declaration of Helsinki.

## Results

Table 1 compares the pre- and post-test scores of the nurses in the HFS and VS groups. The post-test score of the group of nurses exposed to HFS was higher than their pre-test score, with a mean difference of -19.65 and a *p*-value of < 0.001. Both pre-and post-test and pre-and post-OSCE scores had a maximum score of 100 and a minimum score of 0. Moreover, the post-test score of nurses exposed to VS was higher than their pre-test score, with a mean difference of -22.42 and a *p*-value of < 0.001. Thus, both *p*-values affirm a statistically significant difference between the pre- and post-test scores of the two groups.

**Table 1:** Comparing Nurses’ Pre- and Post-test Scores and Pre- and Post-OSCE Scores

*Pre- and Post-test Scores of Nurses*						
**Group**	**Test**	**Mean**	**SD**	**Mean Difference**	*p-value*	**Difference**
Exposed to HFS	*Pre*	75.57	9.32	-19.65	< 0.001	Significant
	*Post*	92.22	5.06			
Exposed to VS	*Pre*	72.88	10.31	-22.42	< 0.001	Significant
	*Post*	95.3	4.88			
*Pre- and Post-OSCE Scores of Nurses*						
Exposed to HFS	Pre	72.19	5.37	23.85	< 0.001	Significant
	Post	96.04	4.19			
Exposed to VS	Pre	72.26	4.04	20.63	< 0.001	Significant
	Post	92.89	4.04			

Furthermore, the post-OSCE scores of nurses in the HFS group were higher than their pre-OSCE scores, with a mean difference of 23.85 and a *p*-value of < 0.001. Furthermore, the post-OSCE scores of nurses in the VS group were higher than their pre-OSCE scores, with a mean difference of 20.63 and a *p*-value of < 0.001. Thus, both *p*-values indicate a statistically significant difference between the pre- and post-OSCE scores of the two groups (refer to Table 1).

Table 2 shows the levels of self-efficacy, self-confidence, and satisfaction acquired by the nurses after their exposure to HFS and VS. Regarding *self-efficacy*, nurses exposed to HFS and VS had mean values of 32.31 and 33.49 out of 40, respectively. Both values indicate a high level of self-efficacy. Similarly, regarding *self-confidence*, nurses in the HFS group had a mean of 4.30, while the VS group had a mean of 4.38, indicating that both groups of nurses acquired a high level of self-confidence. Regarding *satisfaction*, the means were 4.38 and 4.46 for the HFS and VS groups, respectively. These values demonstrate high levels of satisfaction following exposure to HFS and VS.

**Table 2:** Levels of Nurses’ Self-efficacy, Self-confidence, and Satisfaction

Criteria	Groups	Mean	SD	Interpretation
Self-efficacy	HFS	32.31	4.70	High self-efficacy
	VS	33.49	4.21	High self-efficacy
Self-confidence	HFS	4.30	1.10	Confident
	VS	4.38	0.96	Confident
Satisfaction	HFS	4.38	1.13	Satisfied
	VS	4.46	1.02	Satisfied

Table 3 compares the nurses’ groups after exposure to HFS and VS based on acquired self-efficacy, self-confidence, and satisfaction. Out of a total score of 40, the means of acquired self-efficacy of the nurses were 32.31 (HFS) and 33.49 (VS). Out of a total score of five, the means of self-confidence were 4.30 (HFS) and 4.38 (VS), and the means of satisfaction were 4.38 (HFS) and 4.46 (VS). Furthermore, the results revealed that the acquired levels of self-efficacy (*p*-value = 0.13), self-confidence (*p*-value = 0.65), and satisfaction (*p*-value = 0.67) of both groups of nurses were not significantly different between the two modalities. This indicates that exposure to both simulation modalities can enhance and boost the nurses’ levels of self-efficacy, self-confidence, and satisfaction.

**Table 3:** Comparing Nurses’ Acquired Self-efficacy, Self-confidence, and Satisfaction

Assessment	Groups	Mean	SD	Mean Difference	*p*-value	Difference
Self-efficacy	HFS	32.31	4.70	-1.18	0.13	Not significant
	VS	33.49	4.21			
Self-confidence	HFS	4.30	1.10	-0.08	0.65	Not significant
	VS	4.38	0.96			
Satisfaction	HFS	4.38	1.13	-0.08	0.67	Not significant
	VS	4.46	1.02			

## Discussion

This study aimed to evaluate and compare how two simulation modalities (HFS and VS) affected professional nurses’ knowledge, skills, self-efficacy, confidence, and satisfaction following a simulated clinical experience. The study findings confirmed that the post-test and post-OSCE scores of the nurses significantly improved after the simulation exposure compared to their pre-test and pre-OSCE scores. The study revealed that nurses exposed to HFS and VS gained remarkable knowledge and skills. Moreover, nurses exposed to both modalities showed high self-efficacy, confidence, and satisfaction following the simulation experience. Interestingly, both simulation modes had a positive impact on nurses. Furthermore, the nurses’ attitudes improved by effectively interacting and communicating with patients and family members during the simulation. Therefore, SBL can cover the gaps between theoretical knowledge learnt in classrooms and practical skills.

Moreover, we selected nurses with no prior experience in the Oncology unit to eliminate bias from prior knowledge, skills, and experience that might affect the study findings. The effectiveness of the SBL approach using HFS and VS demonstrated strong evidence that SBL can be used to train newly recruited nurses in the Oncology unit as well as experienced oncology nurses on more complex oncologic care scenarios.

The competency of professional nurses is a vital factor influencing the quality of care rendered to patients [6]. Additionally, competent nurses need skills such as decision-making and problem-solving abilities in clinical situations [5]. The simulated environments can expand nurses’ skills and capabilities in specialised tasks without harming patients [20]. Additionally, simulation experience offers high levels of satisfaction in learning, advances professional nurses’ self-confidence [17], and improves their self-efficacy [62]. Sullivan et al. [18] suggested that continuous use of SBL will develop nurses’ knowledge and competence and offer them a safe environment to reflect and resolve their practice-related apprehensions. Furthermore, Cleaver et al. [50] strongly suggest continuous simulation training, using simulation for the refresher courses, and including it as part of the professional development program to prevent skill deterioration and ensure its long-term effectiveness.

Most previous studies have focused on the effect of HFS and VS on nursing students. However, the promising results of this study show that HFS and VS, as simulation modalities and teaching methods, are effective and efficient for nursing students and valuable in enhancing the competency of professional nurses. Moreover, HFS and VS help nurses hone their competencies to practice in real-life clinical environments while providing quality patient care and satisfaction.

### Implications for nursing and health policy

A simulated environment provides immersive and experiential learning. The SBL approach is not only effective in preparing nursing students to practice but also is efficient in improving the knowledge, skills, self-efficacy, confidence, and satisfaction of practising nurses.

The SBL approach will be a potent instrument for improving nurses’ competency and fully developing their sense of expertise. Therefore, developing policies adopting simulation as part of their professional development will ensure patient safety and improve health outcomes.

### Limitations

The study was conducted in only one location in Saudi Arabia. Most participating nurses were women, and only three scenarios were considered. The sample size of each group was *n* = 60 and did not meet the G*power analysis threshold of *n* = 64. Moreover, the effect size for the t-tests was not calculated as the *p*-values were already provided for both practical and statistical significance. Further research must be conducted in multiple settings and locations to reinforce the effectiveness of HFS and VS in improving nurse practitioners’ knowledge, skills, self-efficacy, confidence, and satisfaction in learning.

## Conclusions

This study revealed that HFS and VS training effectively and efficiently advance nurses’ professional competence. Nurses exposed to the HFS and VS showed high levels of acquired knowledge and skills. Furthermore, exposure to these simulation modalities boosted professional nurses’ self-efficacy, confidence, and satisfaction. The simulation method can be a potent instrument for improving nurses’ competency and fully developing their sense of expertise as part of their professional development. Practising in a secured simulated environment lets them commit mistakes freely without endangering patients. Therefore, nurses can learn more from their mistakes and at their own pace, preventing them from committing medical errors in a real environment. This would promote and enhance the safety and quality of patient care.

### Recommendations

Future research must be conducted in multiple settings and locations using multiple simulation scenarios to confirm the findings of this study. Based on the findings, the researcher recommends including an SBL approach using HFS and VS in nurses’ regular professional development training. Assessments must also be conducted following simulation sessions to evaluate nurses’ knowledge and skills gained from the scenario.

### Electronic supplementary material

Below is the link to the electronic supplementary material.

Supplementary Material 1

Supplementary Material 2

Supplementary Material 3

## Data Availability

The data supporting the findings of this study are available from the corresponding author upon reasonable request.

## Abbreviations

AAR: After action review
CSSC: Clinical Skills and Simulation Center
GAS: Gather, analyse, summarise
HFS: High-fidelity simulation
IBM: International Business Machines
OSCEs: Objective structured clinical examinations
SBL: Simulation-Based Learning
SD: Standard deviation
SPSS: Statistical Package for the Social Sciences
VS: Virtual Simulation
