# Supplementary material for: Improvements in practising nurses’ knowledge, skills, self-efficacy, confidence, and satisfaction after a simulated clinical experience of caring for a patient undergoing chemotherapy: a quasi-experimental study
Source: BMC Nurs. 2024 Jan 24;23:66. doi: 10.1186/s12912-024-01727-0 (PMC10807190; doi:10.1186/s12912-024-01727-0)
Supplement: Supplementary file 2 — Supplementary Material 2 [file 12912_2024_1727_MOESM2_ESM.docx]

**OSCE Station No. 1**

**Instruction for the Participants**

This is a six-minutes clinical station. You are requested to perform this task for a duration of 5 minutes, and you are given 1 minute to read the scenario.

**Scenario**

Mrs. Brown is a 67-year-old patient, diagnosed with stage II breast cancer. She was scheduled for her first session of chemotherapy.

**Task**

You are requested to prepare and assess the patient before the chemotherapy session.

**Station No. 1**

**Preparation and Assessment of Patients for Chemotherapy**

**Examiner’s Checklist**

| **No** | **Procedure** | **Done Correctly**  **(3)** | **Done Incompletely**  **(2)** | | **Done Incorrectly**  **(1)** | | **Not Done**  **(0)** |
| --- | --- | --- | --- | --- | --- | --- | --- |
|  | Verify the chemotherapy admission form and prescription form. |  |  | |  | |  |
|  | Introduce yourself. |  |  | |  | |  |
|  | Provide privacy. |  |  | |  | |  |
|  | Perform hand hygiene and put on gloves. |  |  | |  | |  |
|  | Identify the patient correctly. |  |  | |  | |  |
|  | Explain the purpose of the procedure [goals, duration, and side-effects] of chemotherapy administration to the patient and his family. |  |  | |  | |  |
|  | Review patient history including nutritional deficit, fluid restrictions, and known allergies. *** |  |  | |  | |  |
|  | Check patient’s baseline laboratory reports like Hb, WBC, platelet count, LFT, and RFT. *** |  |  | |  | |  |
|  | Obtain written informed consent from patient/family member. *** |  |  | |  | |  |
|  | Check the written order [regimen, date, time, duration, sequence, route, and dose and special instructions] for chemotherapy administration. *** |  |  | |  | |  |
|  | Check the vital signs. |  |  | |  | |  |
|  | Perform a brief physical assessment. |  |  | |  | |  |
|  | Select the chemotherapy drug administration site (IV/PICC line/Chemo-port/oral/feeding tube) and assess for its patency/signs of infection. |  |  | |  | |  |
|  | Prepare all the equipment needed for the procedure. |  |  | |  | |  |
|  | Remove gloves and perform handwashing. |  |  | |  | |  |
|  | Documented patient’s assessment findings. |  |  | |  | |  |
| **Total Score ……. /48** | | | | | | | |
|  | **Global Assessment** | **Satisfactory** | | **Borderline** | | **Unsatisfactory** | |
|  | **Overall Performance: ……. /10** | | | | | | |

Feedback/s (Comment/s):

………………………………………………………………………………………………………………………………………………………………………………………………………………………………………………………………………………………………………………………………………………………………………………………………

Evaluators Name & Signature: …………………………………………………………………………………………….

**Reference**

1. Miglani, G., Kumar, A., Pandey, V., Pareek, P., & Nair, S. K. (2022). Chemotherapy administration checklist for patients receiving chemotherapy: Development and validation. Journal of Education and Health Promotion, 11(1), 397.
2. Newton, S., Hickey, M., Brant, J.M. (2016). Mosby's Oncology Nursing Advisor: A Comprehensive Guide to Clinical Practice 2nd Edition. Elsevier. ISBN-10: ‎ 0323375634.
