# Supplementary material for: Improvements in practising nurses’ knowledge, skills, self-efficacy, confidence, and satisfaction after a simulated clinical experience of caring for a patient undergoing chemotherapy: a quasi-experimental study
Source: BMC Nurs. 2024 Jan 24;23:66. doi: 10.1186/s12912-024-01727-0 (PMC10807190; doi:10.1186/s12912-024-01727-0)
Supplement: Supplementary file 3 — Supplementary Material 3 [file 12912_2024_1727_MOESM3_ESM.docx]

**OSCE Station No. 3**

**Instruction for the Participants**

This is a six-minutes clinical station. You are requested to perform this task for a duration of 5 minutes, and you are given 1 minute to read the scenario.

**Scenario**

Mrs. Brown is a 67-year-old patient, diagnosed with stage II breast cancer. She was scheduled for her first session of chemotherapy.

**Task**

You are requested to manage the patient during and after the chemotherapy session.

**Station No. 3**

**Managing Patients During and After Chemotherapy**

**Examiner’s Checklist**

| **No** | **Procedure** | **Done Correctly**  **(3)** | **Done Incompletely**  **(2)** | | **Done Incorrectly**  **(1)** | | **Not Done**  **(0)** |
| --- | --- | --- | --- | --- | --- | --- | --- |
| *During Chemotherapy* | | | | | | | |
|  | Donning of personal protective equipment including inner gloves, cap, gown, shoe cover, face mask, face shield/goggles, and outer gloves. |  |  | |  | |  |
|  | Ensure that the safety rails of the bed are up to prevent fall. |  |  | |  | |  |
|  | Monitor vital signs and side effects like hypersensitivity, vomiting, extravasation, infiltration, etc. |  |  | |  | |  |
| *After Chemotherapy* | | | | | | | |
|  | Flushing the line with compatible fluid as prescribed. |  |  | |  | |  |
|  | Monitor and record the vital signs and observe the patient for immediate side effects of chemotherapy. |  |  | |  | |  |
|  | Withdraw the IV line, apply dressing at the respective site and observe for bleeding. |  |  | |  | |  |
|  | Doffing of the personal protective equipment, remove shoe cover, outer gloves, face shield/goggles, gown, cap, inner gloves, and face mask. |  |  | |  | |  |
|  | Record the entire procedure with date, time, and side effects. |  |  | |  | |  |
|  | Educate the patient about the use of postchemotherapy medication and precautions to be taken. |  |  | |  | |  |
|  | Ensure the enclosure of discharge summary plan including date of next chemotherapy cycle and laboratory tests required. |  |  | |  | |  |
| **Total Score ……. /30** | | | | | | | |
|  | **Global Assessment** | **Satisfactory** | | **Borderline** | | **Unsatisfactory** | |
|  | **Overall Performance: ……. /10** | | | | | | |

Feedback/s (Comment/s):

………………………………………………………………………………………………………………………………………………………………………………………………………………………………………………………………………………………………………………………………………………………………………………………………

Evaluators Name & Signature: …………………………………………………………………………………………….

**Reference**

1. Miglani, G., Kumar, A., Pandey, V., Pareek, P., & Nair, S. K. (2022). Chemotherapy administration checklist for patients receiving chemotherapy: Development and validation. Journal of Education and Health Promotion, 11(1), 397.
2. Newton, S., Hickey, M., Brant, J.M. (2016). Mosby's Oncology Nursing Advisor: A Comprehensive Guide to Clinical Practice 2nd Edition. Elsevier. ISBN-10: ‎ 0323375634.
